# Prohibited Olympic Medalist with PIEZO1 VUS Who Claims Innocence

**DOI:** 10.3390/ijms252111842

**Published:** 2024-11-04

**Authors:** Balázs Sonkodi, Tímea Kováts, Bence Gálik, Márton Tompa, Péter Urbán, Zsófia Flóra Nagy, Pongrác Ács, Miklós Tóth, Attila Gyenesei

**Affiliations:** 1 Department of Health Sciences and Sport Medicine, Hungarian University of Sports Science, 1123 Budapest, Hungary; timea.kovats@gmail.com (T.K.); tothmik1@hotmail.com (M.T.); 2 Department of Sports Medicine, Semmelweis University, 1122 Budapest, Hungary; 3 Hungarian Swimming Federation, 1007 Budapest, Hungary; 4 Szentágothai Research Center, University of Pécs, 7622 Pécs, Hungary; galik.bence@pte.hu (B.G.); tompa.marton@pte.hu (M.T.); urban.peter@pte.hu (P.U.); pongrac.acs@etk.pte.hu (P.Á.); gyenesei.attila@pte.hu (A.G.); 5 Institute of Genomic Medicine and Rare Disorders, Semmelweis University, 1123 Budapest, Hungary; zsofia.flora@gmail.com; 6 Faculty of Health Sciences, Institute of Physiotherapy and Sport Science, University of Pécs, 7622 Pécs, Hungary; 7 Institute of Laboratory Medicine, Semmelweis University, 1123 Budapest, Hungary

**Keywords:** PIEZO1, SDC2, hematological module, Athlete Biological Passport, mechanotransduction

## Abstract

Competitive athletes are often exposed to extreme physiological loading, resulting in over excessive mechanotransduction during their acute intensive training sessions and competitions. Individual differences in their genetics often affect how they cope with these challenges, as reflected in their high performances. Olympic Medalists are prohibited from providing atypical values in the Hematological Module of the Athlete Biological Passport. Since there was no aphysiological result and the Athlete maintained his innocence, a whole genome sequence analysis was carried out on him and his parents, with the primary focus on the *PIEZO* ion channels encoding gene. *PIEZO1* is known to participate in homeostatic regulation even on a whole-body level, including the regulation of physical performance, circulatory longevity of red blood cells and cell fate determination of mesenchymal stem cells in relation to hydrostatic pressure. However, *PIEZO2* was found to be the principal mechanosensory ion channel for proprioception. These regulatory mechanisms play a pivotal role in mechanotransduction and intensive exercise moments. Interestingly, two variances of uncertain significance of *PIEZO1* were found that may explain the atypical values of the Athlete. Furthermore, two additional variances in *SDC2*, the syndcan-2 encoding gene, were identified in trans position that may influence the crosstalk between *PIEZO2* and *PIEZO1*, with more likely relevance to the detected atypical values. After all, based on the found variances of *PIEZO1* and syndecan-2, it cannot be ruled out that these VUS variants may have caused or impacted the exhibited outlier findings of the ABP Hematological Module of the Athlete.

## 1. Introduction

The Athlete Biological Passport (ABP) is an innovative method of the World Anti-Doping Agency (WADA) and used in the fight against doping. Instead of the direct detection of prohibited substance or method, as is the case in the analytical approach, where an adverse analytical finding provides direct proof of doping practices, ABP looks for longitudinal changes in the level of selected biological markers which cannot be rationalized by physiologic reasons. The Athlete Biological Passport Hematological Module uses the Adaptive Model (a mathematical method based on Bayesian statistics) to individually modify the reference ranges of selected hematologic biomarkers. Since hematopoesis is a finely tuned regulation, influenced by many intrinsic (i.e., genetic) and extrinsic (environmental) factors, the individual reference range, defined by the Adaptive Model, can be very different from the large population-based reference range. Moreover, due to the characteristics of the mathematical model and the often temporary effects of the confounders (and how these influence each other), the limits of the individual reference range are highly dependent on the Athlete’s previous results. While the strict sampling rules of WADA attempt to minimize the effect of several confounding factors, the influence of other confounders, such as the genetic background, cannot be eliminated. Therefore, continuous research in the field is warranted by many scientific reviews [1,2].

Primary biomarkers monitored by the ABP hematological module are hemoglobin concentration (Hgb) and OFFscore, where the latter is a stimulation index calculated from hemoglobin concentration (g/L) and reticulocyte percent (ret%), according to the following formula: OFFscore = Hgb-60√ret%. Atypical Passport Finding (ATPF) is identified if a primary marker falls outside the individual 99% reference range. Thus, an ATPF is a quantitative estimation indicating that the data are outliers of the previous database of the Athlete.

A Passport generating an ATPF has to be reviewed by an expert within seven days, according to the World Anti-Doping Code’s International Standard Results Management, Annex C. 2023. The target of the expert’s evaluation is to provide a qualitative assessment of the outlier data. If the expert’s conclusion is “likely doping” (i.e., the likelihood that the Passport is the result of the use of a prohibited substance or prohibited method outweighs the likelihood that the Passport is the result of normal physiological and pathological conditions), the result has to be re-evaluated by a panel of three experts, including the initial expert. If the unanimous conclusion is, again, “likely doping”, then, and only in this case, the Athlete will be notified of ATPF. From this point on, the legal principle of the presumption of innocence is no longer valid for the Athlete, and they have to prove that the atypical results were not caused by the use of illegal substances or methods.

The case demonstrated in this report is currently at this stage, and it is necessary to prove that the atypical results in the ABP of the Athlete were not caused by the use of any illegal substances or method. Accordingly, the authors were curious to analyze the *PIEZO* genes for any variants that might explain these atypical findings of the Athlete. *PIEZO* proteins are burst-activating, excitatory mechanosensitive ion channels with distinct functional properties. The *PIEZO* proteins were raised as a target for investigation for several reasons. *PIEZO1* was found to contribute to physical performance [3]. Moreover, *PIEZO1* can sense stimuli not only in a spatially restricted manner [4] but on a whole-body level as well [5]. This whole-body mechanostransduction is suggested to be controlled by the nervous system through the *PIEZO2*-*PIEZO1* crosstalk [6]. *PIEZO2* was also found to be the principal mechanosensory channel responsible for proprioception [7], which has pivotal relevance in athletic performance and exercise training sessions. An overexertion-related and often occurring exercise-limiting phenomenon, namely, delayed onset muscle soreness (DOMS), is theorized to evolve due to the autonomously acquired microdamage of *PIEZO2*. Imbalanced control of innate immune cells [8] and exaggerated contractions are symptoms of DOMS, among others, and may have relevance to the presented case when it comes to imbalanced control of reticulocytes or reflexory anaerobic spleen contraction. Tissue repair and regeneration further supports that *PIEZO1* has indeed been implicated in injury response. [9]. Finally, the mechanotransduction system also has its hierarchical order of activation [10], as physical activity is organized hierarchically [11]. *PIEZO* proteins are likely the first activated ion channels in these hierarchies due to their burst-activating feature and homeostatic regulating capacity.

An evolving question of science is whether the *PIEZO* protein could acquire a channelopathy or if channelopathy only exists in inherited or genetically manipulated forms. For years, the scientific consensus rather leaned towards that these proteins cannot be microdamaged in an acquired fashion. The concept of autonomously acquired *PIEZO2* channellopathy was first suggested in 2021 in a strange DOMS related condition [12], but recently, more and more scientists are highlighting the likelihood of acquired microdamage of these *PIEZO* proteins [13,14]. The authors of this paper suspect such microdamage of *PIEZO* ion channels may explain the observed atypical results. Potential underlying *PIEZO* variants with likely functional relevance may increase the susceptibility for these microdamaging events and contribute to individual differences in exercise performance and physiological regulative mechanisms, including even reticulocytes.

Beyond *PIEZO* ion channels contribution to exercise performance and injury, *PIEZO1* is shown to link mechanical forces to red blood cell (RBC) volume through stretch dependence [15] and contribute to the circular longevity of RBCs [16]. Moreover, the gain of function mutation on *PIEZO1* is involved in dehydrating RBC disease xerocytosis [15]. Another important hallmark of *PIEZO1* is that it regulates cell fate determination of mesenchymal stem cells in relation to hydrostatic pressure [17] and these mesenchymal stem cells are known to have the capability to recover even pure RBC aplasia [18].

The current analysis, presented as a case report, was aiming to find any variants of *PIEZO* genes that may have functional relevance to potentially explain the found atypical results of the Athlete based on the aforementioned suspicions. After all, ABP is a smart, sophisticated and powerful tool in our hands in the fight against doping. However, ample of scientists have also warned that ABP has its pitfalls [1,2,19,20] and genetic variants may be considered as such.

## 2. Results

The Athlete had 46 samples included in his Biological Passport between 9 July 2014 and 7 February 2022. However, three samples were rendered invalid later; therefore, these samples were not integrated in the Adaptive Model.

**Figure 1:**
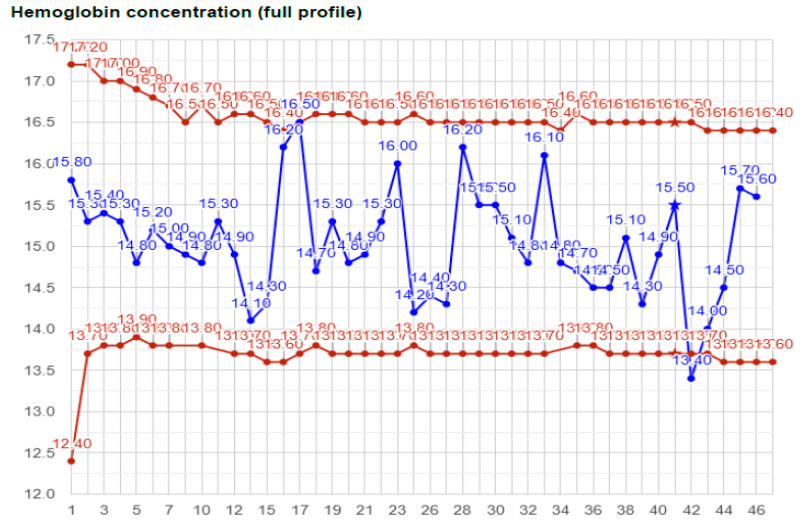
Individual reference range and values of hemoglobin concentration of the Athlete. The Adaptive Model automatically processes data and modifies the population-based upper and lower limit of the reference range, creating an individual reference range (red color) with a specificity of 99% within which marker values (blue) fall, assuming normal physiologic conditions (and typical genetic background). Among 46 samples, the Athlete had two hemoglobin concentration values (#17 and #42) that fell beyond the individual reference range. However, WADA Experts acknowledged that #42 was taken right after an official blood donation, and thus it was taken off the list of the accused results. The value of #17, although falls somewhat outside the individual reference range, it is still well within the normal, population-based reference ranges.

Figure 1 shows the hemoglobin values of the Athlete. Among the 43 samples, 2 were outside the individual reference limit. One of these outliers (sample #42, [Hgb]134 g/L) was due to a legal blood donation, thus, later on, was removed from the list of accused results. Interestingly, none of the results were outside the population-based physiological reference range, or even close to it, i.e., none of these samples would have been considered aphysiological.

**Figure 2:**
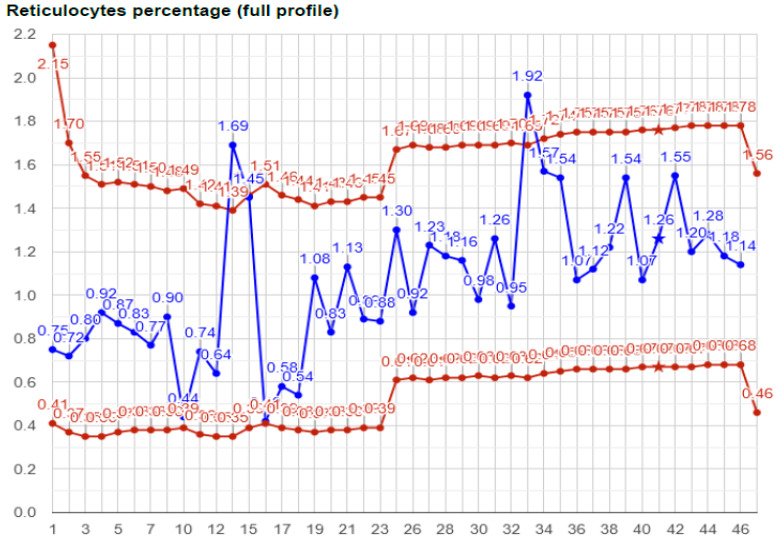
Individual reference range and values of reticulocyte percentage of the Athlete. Among 46 samples, the Athlete had two reticulocyte percentage values (#13 and #33) that fell beyond the individual reference range. However, none of these would be even considered aphysiological according to normal, population-based reference values, even if reference ranges vary among laboratories.

Similarly, reticulocyte percent values in Figure 2 fell outside the individual reference range in two cases out of the included 43 samples. However, these outliers in the ABP model are not even close to the population-based reference limits.

All the ATPFs were extensively studied for possible physiological rationales. The Athlete was thoroughly interrogated for possible extrinsic confounding factors such as training volume and intensity or the presence or lack of high-altitude training, as well as any health issues that might have altered his blood parameters. In a comprehensive and overly meticulous evaluation, detailed physiological explanations supported by scientific references for all the outliers were provided to the experts of the World Anti-Doping Agency. Unfortunately, the dimension of the physiological reasoning is beyond the extension of this case report and will be published separately. Since all the ATPFs were within the physiological range and could be well explained physiologically, yet still represented outliers by a mathematical model, we looked for possible intrinsic confounders; namely, a whole exome trio sequencing was completed with special focus on the PIEZO genes.

### 2.1. WES Trio Coverage and Sequencing Quality

The average coverage for the proband was 181x, for the father it was 151x and for the mother it was 156x. The uniformity at 20x was 98.69% in the proband, 98.67% in the father, and 98.46% in the mother. The on-target read count was 74,535,114 in the proband, 64,225,505 in the father, and 64,708,040 in the mother.

### 2.2. Genomic Variants in the Proband

A total of 85,058 high-quality variants were identified in the proband. We investigated the variants after applying read depth and qenotype quality filtering (see Method xy section,). A total of 32 *PIEZO1* and 49 *PIEZO2* variants were identified, while only two *SDC2* variants were found. Next, we further filtered the variants to remove those that are frequent in the general population (see Method xy section,), which resulted in a total of 3758 variants. Of these 3758 variants, only two *PIEZO1* and two *PIEZO2* variants, and none of the *SDC2* variants remained. Both variants of *PIEZO2* are deep intronic. One variant is in a heterozygous state, while the other one is in a homozygous state. The homozygous variant was previously registered as a benign variant in ClinVar (ID:1180929) (Table 1). The two variants of *PIEZO1* are 266 base pairs apart from each other, in trans position. One component of the compound heterozygous alterations is a 24-base pair in-frame deletion coming from the mother: NM_001142864.4:c.2230 _2253del, p.(His744_Glu751del) and the other one is a single nucleotide substitution in an intronic region coming from the father: NM_001142864.4:c.2180 + 108C > T, p. (Table 1). No publications involving these two variants or other variants in the same domain were found in the literature. Their clinical significance remains uncertain.

## 3. Case Presentation

A male Olympic medalist was sent a letter by the Athlete Biological Passport Joint Expert Opinion on 28 September 2022. The letter stated that his atypical hematologic results were likely attributed to blood manipulation and it was unlikely the result of any other cause. The Athlete is maintaining his innocence; specifically, he is stating that he has not used any blood manipulation and refrained from EPO usage. In a defense of the Athlete, a thorough physiological explanation of the hematologic results was submitted to the WADA Expert Panel, however, this reasoning was accepted only partially and the prohibitive ruling was upheld on 11 January 2023. The Athlete further maintained his innocence and appealed to the Court of Arbitration for Sport, Lausanne, Switzerland, where his case is ongoing.

In a random blood test of the Athlete’s sedentary father, hemoglobin, reticulocyte and OFFscore values gave atypical results: Hgb 157, ret% 2.41, OFFscore 63. It is very well known that athletes’ intrinsic characteristics, such as ethnicity and genetic background, produce variations in the changes of hematological parameters [2], underpinning the reviews written on the ABP of those always emphasize the importance of continuous research on potential confounders [1,2]. Atypical results from the Athlete’s sedentary father increased the authors’ suspicion that the father’s genetic background may have contributed to the Athlete’s values falling outside the expected range.

Correspondingly, the authors’ goal was to detect any non-pathogenic genetic variances in the Athlete’s genome that might answer the aforementioned scientific puzzle based on the suspicion elaborated in the current introduction, not to mention the above mentioned familial one. Thus, the authors conducted a comprehensive genetic testing on the Athlete and his parents (whole-genome trio sequencing). We aimed first to examine the encoding genes of *PIEZO1* and *PIEZO2* ion channel proteins.

## 4. Materials and Methods

### 4.1. Analysis of the Samples in Three Rounds

To determine phase of the potential variants associated with the putative phenotype and mechanism of inheritance, a trio WES study was performed. In the trio analyses, variants were assessed simultaneously in the proband, the father and the mother. First, we focused only on the *PIEZO1*, *PIEZO2* and *SCD2* gene variants. Second, we performed a virtual mini panel-based analysis with genes that could potentially interact with the *PIEZO1* and *PIEZO2* genes. Third, we searched for pathogenic, likely pathogenic, or variants of unknown significance (VUS) variants that could potentially be associated with the putative phenotype in addition to the genes previously analyzed.

### 4.2. Sample Preparation and Quality Check

The nucleic acid concentration and fragmentation of the samples were checked after nucleic acid extraction. Subsequently, DNA was enzymatically fragmented, the ends were repaired, Illumina-specific adapters were ligated, and the libraries were amplified. Nucleic acid isolation was performed using an Omega Biotek Mag-Bind^®^ Blood & Tissue DNA HDQ 96 Kit (Omega Bio-tek, Inc., Norcorss, Georgia). DNA concentration was measured by using ThermoFisher Qubit 4.0 (Invitrogen, Carlsbad, CA, USA) and fragmentation was evaluated by Agilent TapeStation 4200 (Agilent Technologies, Santa Clara, CA, USA). A short read sequencing library of DNA samples was prepared using xGen DNA Library Prep Kit EZ reagents (Integrated DNA Technologies, Inc., Coralville, IA, USA), and the target regions were enriched with xGen hybridization assays. The quality of the prepared library was checked and sequencing was performed on Illumina NovaSeq X Plus platform (Illumina Inc., San Diego, CA, USA) with 2 × 150 base pair reads.

### 4.3. Bioinformatic Analysis

Raw sequencing data, including base calls and sample sorting, were generated with bcl2fastq (v2.20.0.422). Single nucleotide variants (SNVs) and short insertions and deletions (INDELs) were analyzed by custom-developed data analysis workflow. As a first step, raw data quality control was performed using FastQC (v0.11.9) software. Adapters and poor-quality bases and sequences were removed using fastp (v0.21.0). The filtered, high-quality sequences were aligned to the human reference genome (hg19/GRCh37) using the bwa mem algorithm (v0.7.17). The BAM file modifications (alignment, sequence metadata addition, index generation, duplicate removal) were performed by Picard Tools software package (v2.23.3). Mapping statistics were calculated using Picard Tools and Qualimap (v2.2.1). Before the variant call, the baseline quality values were recalibrated using the dbSNP155 database of known mutations and the GATK BSQR module (v4.1.4.1). Variant calling and filtering were performed using different GATK modules based on a file containing genomic coordinates of target genes and regions. The sensitivity for the detection of SNVs and INDELs was 99% with minimum coverage (≥20).

### 4.4. Variant Filtering and Analysis

VarSeq software version 2.6.1 (Golden Helix, Bozeman, MT, USA) was used to annotate, filter and assess the clinical significance of the variants. First, we filtered out low-quality variants with lower than 20 read depths and 30 genotypes quality. Second, variants with high frequency in population databases were removed. We considered the variant common in the population with equal or higher than 1% alternative allele frequencies (in gnomAD Exome Variant frequencies 2.1.1), equal or higher than 1% minor allele frequencies (in NHLBI ESP6500 SI-V2-SSA137 Exome Variant Frequencies 0.0.30) and with an entry in dbSNP (dbSNP Common 155, NCBI) database. In silico pathogenicity, predictor softwares (e.g., Polyphen-2, SIFT, PhyloP, GERP++,CADD, REVEL, Splice AI) were used and the available up-to-date scientific literature overview was performed to remove nonsignificant variants. Finally, variants with loss of function (LoF), missense and other predicted effects (RefSeq Genes 105.20220307, NCBI) of possible clinical significance based on the ACMG classification criteria were retained, while alteration with benign or likely benign entry in the ClinVar database were removed. Variant manual classification was performed according to the ACGS 2024 guidelines [21]). Identified variants were reported by using the consensus-based Human Genome Variation Society (HGVS) nomenclature, exact chromosomal position, zygosity, mode of inheritance and variant support/total read numbers. The final report included the most relevant variants associated with the presumed phenotype based on manual interpretation, scientific literature and available clinical data.

## 5. Discussion

Two *PIEZO1* VUS-s were found in the proband which were later confirmed to be in a trans position. The maternally inherited *PIEZO1* variant, NR_103774.1:n.269 + 2539_269 + 2562del, NM_001142864.4:c.2230_2253del) is a non-frameshift in-frame deletion of 8 amino acids. The variant is located in a repetitive region and the region is known to have a sequential bias towards acidic residues. Although the loss of the 8 amino acids may not be pathogenic, it certainly can have functional implications to the working of the *PIEZO1* channel since the variant disrupts the physiological sequential bias of the region. The paternally inherited variant (NR_103774.1:n.269 + 2800G > A, NM_001142864.4:c.2180 + 108C > T) is located downstream of the 16th exon of the gene in a deep intronic position and leads to cytosine-thymine change. Deep intronic variants are not well-understood yet and might influence the splicing process of the mRNA. The interplay of these variants has not yet been explored but our group stands by the possible functional consequence of these variants.

Indeed, the exploration and rapid increase in our knowledge in regards to the principality of the *PIEZO* channels in homeostatic regulation [6] and in proprioception [7] was recognized by the awarding of the Nobel Prize to Ardem Patapoutian in October, 2021. He reported the discovery of the *PIEZO* ion channels as late as only in 2010 [22]. The exact topology and function of these evolutionarily conserved large transmembrane mechanosensitive channels, in fact, the largest currently known, are far from entirely explored [23]. However, emerging scientific findings are rapidly increasing our understanding.

*PIEZO* ion channels are the fastest-activating mechanosensory ion channels instigating proprioceptive signaling [24], not to mention that *PIEZO2* is the principal ion channel responsible for proprioception [7]. Indeed, it should be considered that mechanotransduction is the conversion of physical cues into biological and chemical signals, and from a cellular to whole-body level. An important consideration is that mechanotransduction is a hierarchical system [10]. *PIEZO1* channels, as one of the fastest-activating ones during mechanotransduction, sense and respond not only on spatially restricted manner [4], but at the whole-body level as well in order to enhance performance and reset homeostasis [5], which is suggested to be controlled by the nervous system through the *PIEZO2*-*PIEZO1* crosstalk [6]. Another indication that one recent study found is that human physical activity is also organized on a high-level of hierarchy [11] like the aforementioned mechanostransduction system [10]. *PIEZO* channels likely play a principle and initiating role in the regulation of these hierarchies.

In addition, endothelial *PIEZO1* could regulate osmolarity and water outflow dynamics in correlation with exercise intensity with special relevance under fatigue-induced somatosensory hyperexcitability [25]. These characteristics and somatosensory feedback are extremely important in acute intensive exercise activities. In addition, the microdamage of *PIEZO,* due to over-excessive mechanotransduction, or the acquired *PIEZO* channelopathy, is considered to be a gateway between physiology and pathophysiology, hence the breach of the limits of homeostatic tissue remodeling [26,27]. Repeated damage to these ion channels could also carry relevance in the chronification of the initial microdamage [12,26].

Accordingly, it has been shown that *PIEZO1* is the ion channel linking mechanical forces to RBC volume by exhibiting robust calcium entry in response to mechanical stretch [15]. Genetic variants of *PIEZO1* do matter in this regulation since gain-of-function mutation on *PIEZO1* leads to the dehydration of RBC disease xerocytosis [15]. Moreover, *PIEZO1*-mediated intracellular calcium entry above a certain threshold in the circulation could lead to early or late hyperdense collapse of RBCs, followed by delayed density reversal, hence affecting the circulatory longevity of human RBCs [16]. It has been theorized that *PIEZO* channels could go through acquired channelopathy in an acute transient [12], chronic [12] and irreversible [28] fashion and this microdamage could be explained by the conformational changes of the cellular membrane nanodomains around the *PIEZO* channels and its auxiliary proteins [27], leading to subthreshold leakage calcium currents [12,29]. Correspondingly, this could possibly explain the early or late hyperdense collapse of RBCs followed by delayed density reversal, in an analogous mechanism as is hypothesized for delayed onset muscle soreness [6,12]. In brief, *PIEZO* channelopathy could be considered the breach of homeostatic tissue remodeling [27] that could be possibly applied to circulatory homeostatic hemoconcentration and hemodilution regulation as well [25].

Since *PIEZO2* channelopathy is also suggested to be a principal transcription activator, underlying genetic variants could be revealed [30] and could have relevance for individual differences. Hence, mutations in *PIEZO* channels do count, and not only in a disease-causing manner, like in xerocytosis, but in minor variances of uncertain significance (VUS), which could be reflected in a non-disease causing manner leading to individual differences in response to acute intensive exercise performances.

Another important consideration is that *PIEZO1* regulates cell fate determination of mesenchymal stem cells in relation to hydrostatic pressure [17]. Correspondingly, these mesenchymal stem cells are even capable of recovering pure RBC aplasia without any side effects [18].

Scientists suspect cross-talk between *PIEZO1* and *PIEZO2* [26,31,32], and this cross-communication is later suggested to be a cross-frequency coupling through Huygens synchronization [27,33]. This theoretical link has been depicted recently [27,33]. The impairment of this cross-talk could lead to a switch in proprioceptive wiring, metabolism and insulin resistance, but it also activates transcription in a principle way and the inflammatory/gateway reflex, not to mention induces autonomic dysregulation [27,30]. An important consideration is that the impairment of this cross-talk could arise from both directions, from *PIEZO1* channelopathy and *PIEZO2* channelopathy as well [26]. *PIEZO2* is associated with reflex mechanisms [12,28] and might play a role in the inducement of reflexory anaerobic spleen contraction under intense exercise [25]. It is noteworthy that the consequence of *PIEZO2* channelopathy is not only the impaired *PIEZO2*-*PIEZO1* cross-talk but the inducement of impaired reflexes [29] and exaggerated contractions [12,29]. This could explain the transient dysregulation of heamoconcentration and hemodilution under intense exercise [25] and not only the aforementioned reflexory anaerobic spleen contraction.

Moreover, an important protein family and its encoding genes might play a pivotal role in the *PIEZO2*-*PIEZO1* cross-talk, namely syndecans [27,33]. These glycoproteins may act like proton-collecting antennas with their negative charge contributing to ultrafast long-range proton-based signaling, but they could also loose this capability when it comes to shedding under excessive mechanostransduction [27]. Furthermore, Syndecan-2 has a known role in hematopoietic stem cells regulation [34]; hence, it may further influence the above-mentioned *PIEZO1* contribution to the regulates of cell fate determination of mesenchymal stem cells. An important finding is that we identified two variants in *SDC2*, the syndcan-2 encoding gene, in trans position that may impact the aforementioned *PIEZO2*-*PIEZO1* crosstalk, in addition to hematopoietic stem cells regulation.

Additionally, the Athlete has experienced a remarkable weight-gain since the prohibitory action, namely up to 30 kg, which is a significant 37.5% increase. An important scientific finding is that *PIEZO1* plays a central regulatory role in insulin sensitivity, glucose metabolism and energy expenditure in obesity [35]. Furthermore, mechanosensitive *PIEZO* channels also play a role in the mediation of pathophysiological changes in the respiratory system, more specifically in asthma [36], which is part of the Athlete’s medical history.

## 6. Conclusions

The current authors cannot rule out that the detected *PIEZO1* and syndecan-2 VUS variants may have caused or impacted the exhibit outlier findings of the ABP Hematological Module of the Athlete. The authors strongly urge the scientific community to conduct further research and functional testing of these detected variants and possibly others. Furthermore, they appeal to WADA to develop a method for taking genetic variants, like the reported, ones into consideration, even if full functional results or direct links to these genetic findings are not yet available. This is important because *PIEZO*-related research is on the rise in an unprecedented pace, and related findings have been emerging that could foreseeably devalue WADA’s highly respected fight against doping.

## Figures and Tables

**Table 1:** The table shows bioinformatic parameters of the two identified PIEZO1 and two PIEZO2 variants. In addition, the SDC2 variants are also presented. HGVS = Human Genome Variation Society, VAF = Variant allele frequency, AD = Allelic depths, DP = Read depths.

Variant Info		RefSeq Genes 105.20220307, NCBI					Proband					Mother			Father		
Chr:Pos	Ref/Alt	Gene Names	Sequence Ontology	Effect	HGVS c.Dot	HGVS p.Dot	VAF	AD	DP	Zygosity	Inherited from	VAF	AD	DP	VAF	AD	DP
16:88800390	CTCCTCCTGCTGCTGCTGCTGATG/-	PIEZO1	Inframe deletion	Missense	NM_001142864.4: c.2230_2253del	NP_001136336.2: p.His744_Glu751del	36%	136.75	211	Heterozygous	Mother	33%	102.51	153	-	-	-
16:88800656	G/A	PIEZO1	Intronic variant	Other	NM_001142864.4: c.2180 + 108C > T	-	47%	20.18	38	Heterozygous	Father	-	-	-	44%	23.18	41
18:10762436	A/G	PIEZO2	Intronic variant	Other	NM_022068.4: c.3174 + 62T > C	-	40%	28.19	47	Heterozygous	Mother	52%	30.32	62	-	-	-
18:10763218	T/C	PIEZO2	Intronic variant	Other	NM_022068.4: c.2872-122A > G	-	100%	0.39	39	Homozygous	Both	100%	0.61	61	100%	0.36	36
8:97506373	C/G	SDC2	5 prime UTR variant	Other	NM_002998.4: c.-127C > G	-	43%	13.10	23	Heterozygous	Father	-	-	-	24%	16.5	21
8:97605800	C/T	SDC2	Synonymous variant	Other	NM_002998.4: c.153C > T	NP_002989.2: p.Tyr51=	47%	75.67	142	Heterozygous	Ambiguous	37%	71.41	112	51%	62.64	125

## Data Availability

The data presented in this study are available on request from the corresponding author.
